# Unraveling proteome changes and potential regulatory proteins of bovine follicular Granulosa cells by mass spectrometry and multi-omics analysis

**DOI:** 10.1186/s12953-019-0152-1

**Published:** 2019-10-25

**Authors:** Shuning Hou, Qingling Hao, Zhiwei Zhu, Dongmei Xu, Wenzhong Liu, Lihua Lyu, Pengfei Li

**Affiliations:** 1 College of Life Science, Shanxi Agricultural University, Taigu, 030801 Shanxi China; 2 College of Animal Science and Technology, Shanxi Agricultural University, Taigu, 030801 Shanxi China

**Keywords:** Bovine, Follicle, Proteomic analysis, Multi-omics data analysis, Label-free

## Abstract

**Background:**

In previous study, we performed next-gene sequencing to investigate the differentially expressed transcripts of bovine follicular granulosa cells (GCs) at dominant follicle (DF) and subordinate follicle (SF) stages during first follicular wave. Present study is designed to further identify the key regulatory proteins and signaling pathways associated with follicular development using label-free liquid chromatography-tandem mass spectrometry (LC-MS/MS) and multi-omics data analysis approach.

**Methods:**

DF and SF from three cattle were collected by daily ultrasonography. The GCs were isolated from each follicle, total proteins were digested by trypsin, and then proteomic analyzed via LC-MS/MS, respectively. Proteins identified were retrieved from Uniprot-COW fasta database, and differentially expressed proteins were used to functional enrichment and KEGG pathway analysis. Proteome data and transcriptome data obtained from previous studies were integrated.

**Results:**

Total 3409 proteins were identified from 30,321 peptides (FDR ≤0.01) obtained from LC-MS/MS analysis and 259 of them were found to be differentially expressed at different stage of follicular development (fold Change > 2, *P* < 0.05). KEGG pathway analysis of proteome data revealed important signaling pathways associated with follicular development, multi-omics data analysis results showed 13 proteins were identified as being differentially expressed in DF versus SF.

**Conclusions:**

This study represents the first investigation of transcriptome and proteome of bovine follicles and offers essential information for future investigation of DF and SF in cattle. It also will enrich the theory of animal follicular development.

**Electronic supplementary material:**

The online version of this article (10.1186/s12953-019-0152-1) contains supplementary material, which is available to authorized users.

## Introduction

Mono-ovulatory species such as cattle undergoes 2–3 follicular wave during one estrous/menstrual cycle. In each follicle wave, a group of follicles begin to grow and only one of them get selected to become DF and cause regression of remaining group of follicles known as SF [1]. In general, only the DF eventually ovulates, the meiotically competent oocyte and transform into progesterone secreting CL gland. Both of these events are critically important as the quality of ovulated oocyte determines the success of early embryo development and secretory CL progesterone establish and maintain the pregnancy in cattle. Previous research indicated that follicular development is closely associated with the estrous behavior, estrous cycle, oocyte competency and embryo survival rate [2]. However, any abnormality or reproductive disorders associated with follicular development can be a major cause of infertility in cow as well as human [3]. Therefore, it is critical to understand the molecular mechanism regulating normal follicular development in cattle. Several studies have been performed in the past to understand the physiological regulation of follicular development modulation and ovulation mechanism, such as gonadotrophin interacts with follicular growth factor [4–6], the relationship between apoptosis of follicle GCs and follicle atresia [7–10], the influence of hormone such as estrogen and progesterone [11], the role of growth factor such as vascular endothelial growth factor (VEGF) and insulin-like growth factors 1 (IGF-1) [12–14], the effect of concentration of reactive oxygen [15]. In addition, transcriptome of follicles at different developmental stage have also been analyzed to understand the molecular mechanism regulating bovine follicular development [16]. However, characterizing the functionally available proteins resulting from mRNA translation would provide novel insight into the signaling pathways and biomarkers of follicular developmental process in cattle. As per our knowledge, this is the first study where we characterized the proteome of bovine GCs at DF and SF in first follicular wave and performed an integrated analysis of protein abundance and transcript level to reveal the molecular regulation of bovine follicular development.

## Materials and methods

### Animal care

All animal procedures were implemented in strict accordance with the principles outlines in “Guide for the care and use of Laboratory Animals” by National Institutes of Health.

### Samples collection

Three Holstein dairy cows selected for estrus synchronization with prostaglandin F2α (PGF2α), follicular diameter and growth were observed and recorded by daily ultrasonography, ovaries were removed from cows when the largest follicle appeared and the growth rate was significantly higher than growth rates of other follicles, and the diameter of largest follicle is 8.5 mm, the largest follicle and second largest follicle were collected and considered as DF and SF, respectively. GCs from DF and SF were isolated and stored at − 80 °C until used for further experiment.

### Extraction and digestion of total proteins

GCs were lysed by ultrasonication and homogenization in cold extraction buffer (7 M urea, 40 mM Tris-HCl, 1% dithiothreitol, 1 mM EDTA, 1% Protease Arrest). The lysate was centrifuged at 4 °C for 40 min and supernatants were collected and filtered for protein quantification using Bradford Coomassie® Brilliant Blue G-250 method. After quantification, 50 μg of protein from each sample were denatured using 1 M urea and incubated for 10 min at room temperature, followed by addition of 10 mM dithiothreitol and incubation for 1 h at 56 °C. Proteins were then alkylated with 55 mM iodoacetamide in the dark for 40 min at room temperature and were further subjected to digestion with 1 μg trypsin for 16–17 h at 37 °C [17].

### Label-free liquid chromatography-tandem mass spectrometry (LC-MS/MS)

The content of each peptide was determined and quantified by Capillary High Performance Liquid Chromatography (Eksigent 425, AB SCIEX) and label-free mass spectrometry [18]. (A) H_2_O + 0.1% formic acid and (B) acetonitrile+ 0.1% formic acid, were consisted of the mobile phase. Desalting of the samples was performed online using a reversed-phase C18 trapping column (0.1 mm internal diameter, 20 mm length, 3 μm particle size; Waters). The peptides were then separated using nano-column (0.75 mm internal diameter, 150 mm length, 5 μm particle size; Waters) at 0.3 μL/min. Peptides were eluted from the column using the following gradient: 5–80% B in 110 min, 80–5% B in 0.1 min, maintained at 5% for 120 min and then back to initial conditions. Mass spectrometer (Q-Exactive; Thermo Scientific, USA) was connected to the liquid chromatography apparatus to detect the eluted peptides. The separated peptide fragments were identified by mass spectrometer operated in positive ion mode with electrospray ionization and collision-induced dissociation (CID). Full-scan MS spectra (350–1750 m/z) was acquired at a resolution of 70,000 with an automatic gain control (AGC) target value of 3e6 by electrospray ionization. The full-scan maximum injection time was 20 ms (millisecond), and the dynamic exclusion was set to 25.0 s. CID spectra were acquired at a resolution of 17,500 with an AGC target value of 2e5 and a maximum injection time of 80 ms. The isolation window was set to 2.0 m/z.

### Data analysis

Raw data were imported into Expressionists software (Proteome Discover 2.0) for processing [19], the quantification was performed based on the peak intensities of the report ions of the only unique peptides in the MS/MS Spectra. MS/MS spectra were searched against the Uniprot-COW FASTA database (version 2017–10) with the following mascot parameters [20, 21]: peptide mass tolerance for ±15 ppm and fragment mass tolerance for 20 mmu. Trypsin was used as the protein-cleaving enzyme, and the two missed cleavages were accepted. Carbamidomethylation of cysteine was designated as a fixed modification, and oxidation of methionine, acetylation on protein N-term were selected as variable modifications. The peptide confidence was high, and the peptide length was set to > 4. The peptide false discovery rate (FDR) was set to ≤0.01 [22].

### Bioinformatics analysis

Gene ontology (GO) assignments (http://www.geneontology.org) were used to classify the functions of the differentially expressed proteins (fold change ≥2), and could be categorized into three main categories: biological process, cellular component and molecular function. The most important biochemical metabolic pathways and signal transduction pathways were identified by PANTHER and KEGG PATHWAY (http://www.genome.jp). Cluster of Orthologous Groups (COG) of proteins analysis was used to cluster the original functions of proteins (http://www.pantherdb.org).

### Multi-omics data analysis

Venn diagram and heatmap were used as tools for multi-omics correlation analysis. Overlap of differentially expressed genes between transcriptome and proteome was displayed by venn diagram. As an intuitive figure, a heatmap could visualize the matrix data by representing individual values with different colours.

### Western blotting for validation

We have validated three proteins (OGN, ROR2, HSPB1) and experiment details are available on 《Proteomic characterization of bovine granulosa cells in dominant and subordinate follicles》 [23].

## Results

### Top 30 highly differentially expressed proteins in DF and SF

The top 30 highly differentially expressed proteins in GCs of bovine DF and SF are shown (Table 1), including all 26 upregulated proteins and 4 downregulated proteins.

**Table 1:** Top 30 highly differentially expressed proteins in DF and SF

uniprot	protein name	DF mean	SF mean	DF/SF	*P* value
A5D9E8	OGN	23,344,333,333	18,603,000,000	12.034	0.041
P01045	KNG2	17,741,666,667	16,726,333,333	6.610	0.018
A6QLS5	MGC143209	15,843,333,333	12,345,666,667	5.764	0.041
Q3SZZ9	FGG	8,715,066,667	9,154,200,000	5.624	0.018
F1MAV0	FGB	7,831,366,667	6,321,800,000	5.584	0.019
P62739	ACTA2	5,331,333,333	5,513,366,667	5.382	0.011
A7YWQ4	SNTB2	4,380,900,000	5,185,966,667	5.239	0.036
A5PJE3	FGA	2,550,033,333	4,518,066,667	5.053	0.007
E1BGS2	ACAD10	2,491,466,667	4,378,000,000	4.894	0.001
E1BDU0	NUP107	2,489,033,333	4,180,776,667	4.084	0.004
G3X6T9	FLOT2	2,103,033,333	4,086,033,333	3.533	0.028
F1 N102	C8B	1,509,833,333	3,429,200,000	3.495	0.041
G3X7I5	4 SV	1,368,263,333	3,154,466,667	3.398	0.012
F6RB08	KIF17	1,288,343,333	3,135,646,667	3.375	0.046
F1MTK4	LPCAT1	1,208,773,333	2,667,900,000	2.924	0.028
F1N0F2	SIAE	1,094,433,333	2,598,433,333	2.878	0.001
A8YXZ2	C8G	1,036,296,667	2,314,433,333	2.799	0.050
G3MZ95	FHL1	966,820,000	2,305,566,667	2.704	0.047
A1L5A6	ADRM1	906,270,000	1,791,266,667	2.565	0.036
P41976	SOD2	761,470,000	1,637,366,667	2.539	0.002
D3K0R6	ATP2B4	750,666,666.7	1,625,866,667	2.427	0.004
E1BHR3	HDGFRP3	592,620,000	1,380,866,667	2.373	0.024
F1 MJ71	COL4A4	561,006,666.7	1,348,310,000	2.238	0.002
Q3ZBT5	STX7	525,786,666.7	1,292,716,667	2.156	0.002
E1BMM0	NCBP1	511,866,666.7	1,217,820,000	2.151	0.015
F6RQK3	GSTZ1	511,250,000	1,204,496,667	2.116	0.009
F1MUX6	GSTM3	509,750,000	1,165,810,000	0.496	0.038
E1BHJ0	3 SV	472,130,000	1,121,570,000	0.494	0.011
F1MNG3	RAC1	470,410,000	1,030,903,333	0.485	0.031
Q3T0F4	RPS10	468,846,666.7	1,004,480,000	0.484	0.026

### Go analysis

**Fig. 1:**
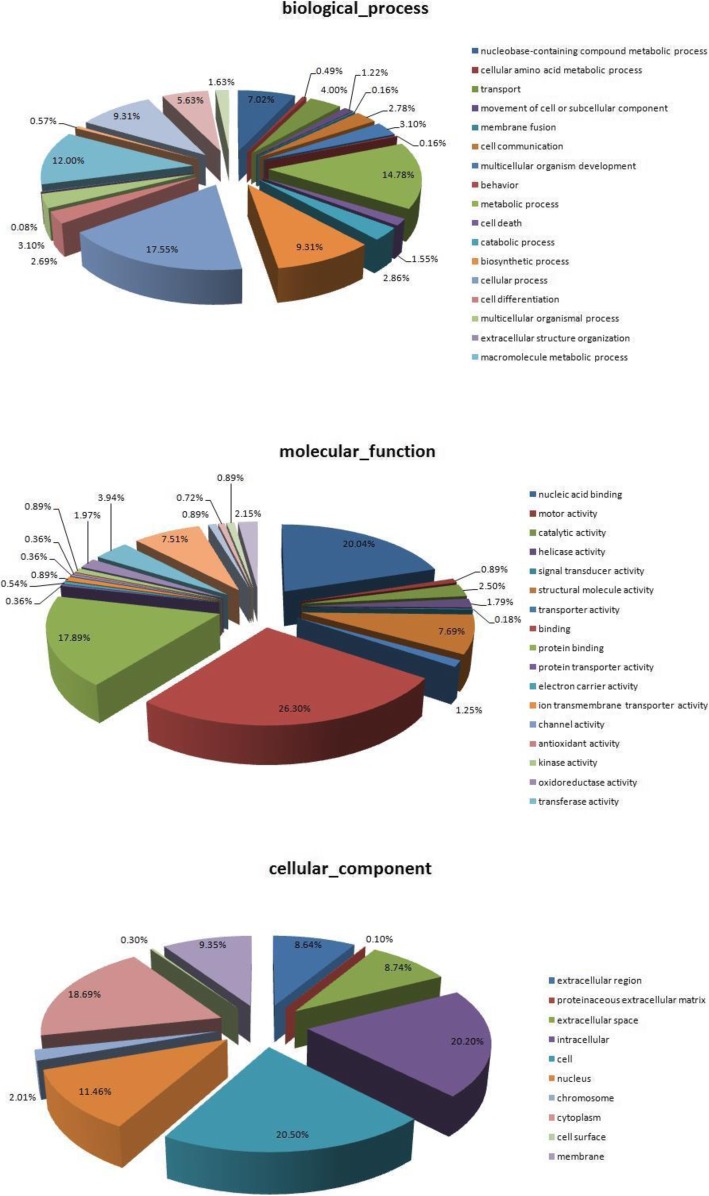
Differentially expressed proteins GO annotation. Classification of the 259 proteins in GCs of DF and SF in cattle based on the gene ontology annotation, respectively. 1–1: biological processes, 1–2: molecular function, 1–3: cellular components. Numbers in percentages (%) correspond to the percentage of respective GO category in the pie chart

**Fig. 2:**
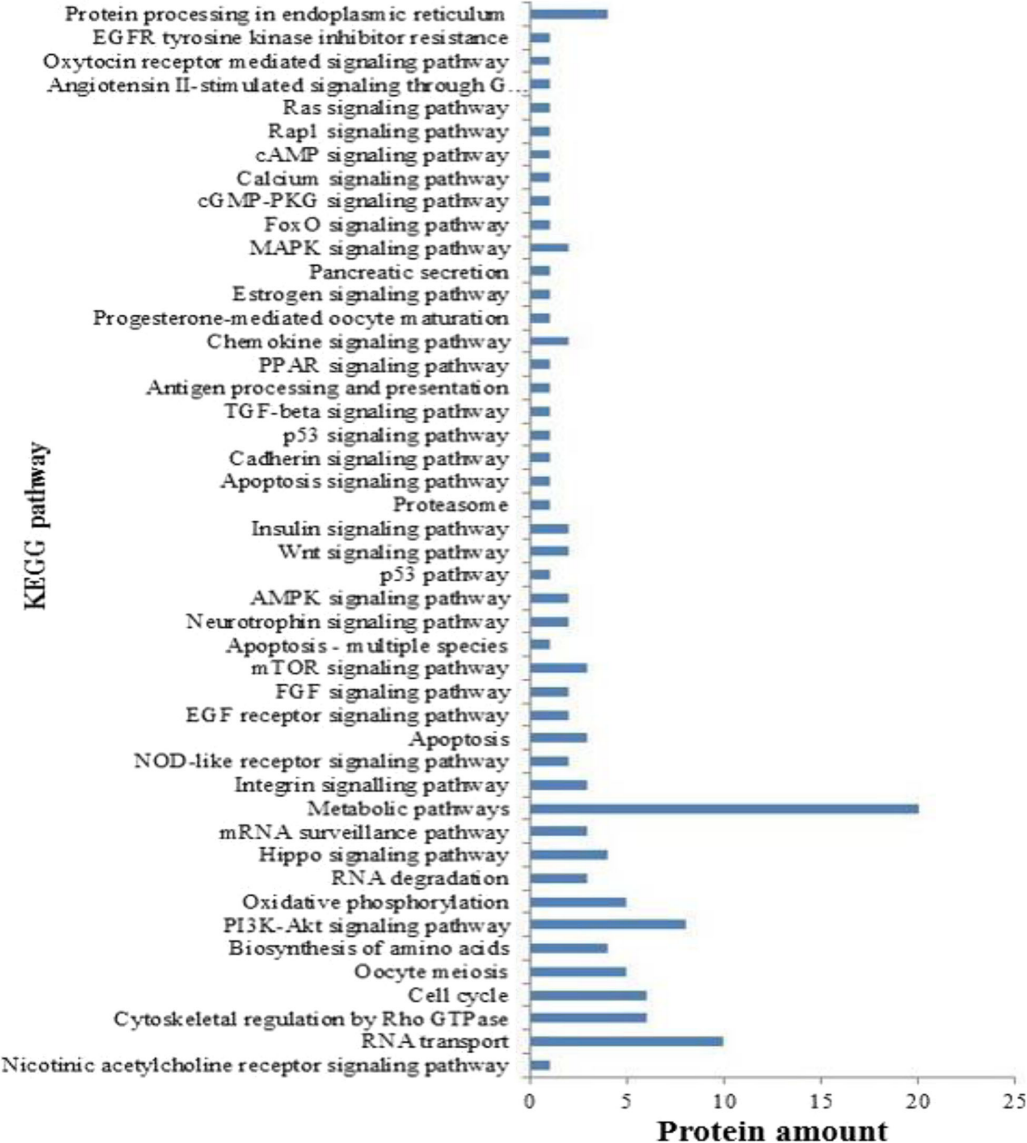
Differentially expressed proteins KEGG pathways analysis. x-coordinate as protein amount, y-coordinate as signaling pathway names

**Fig. 3:**
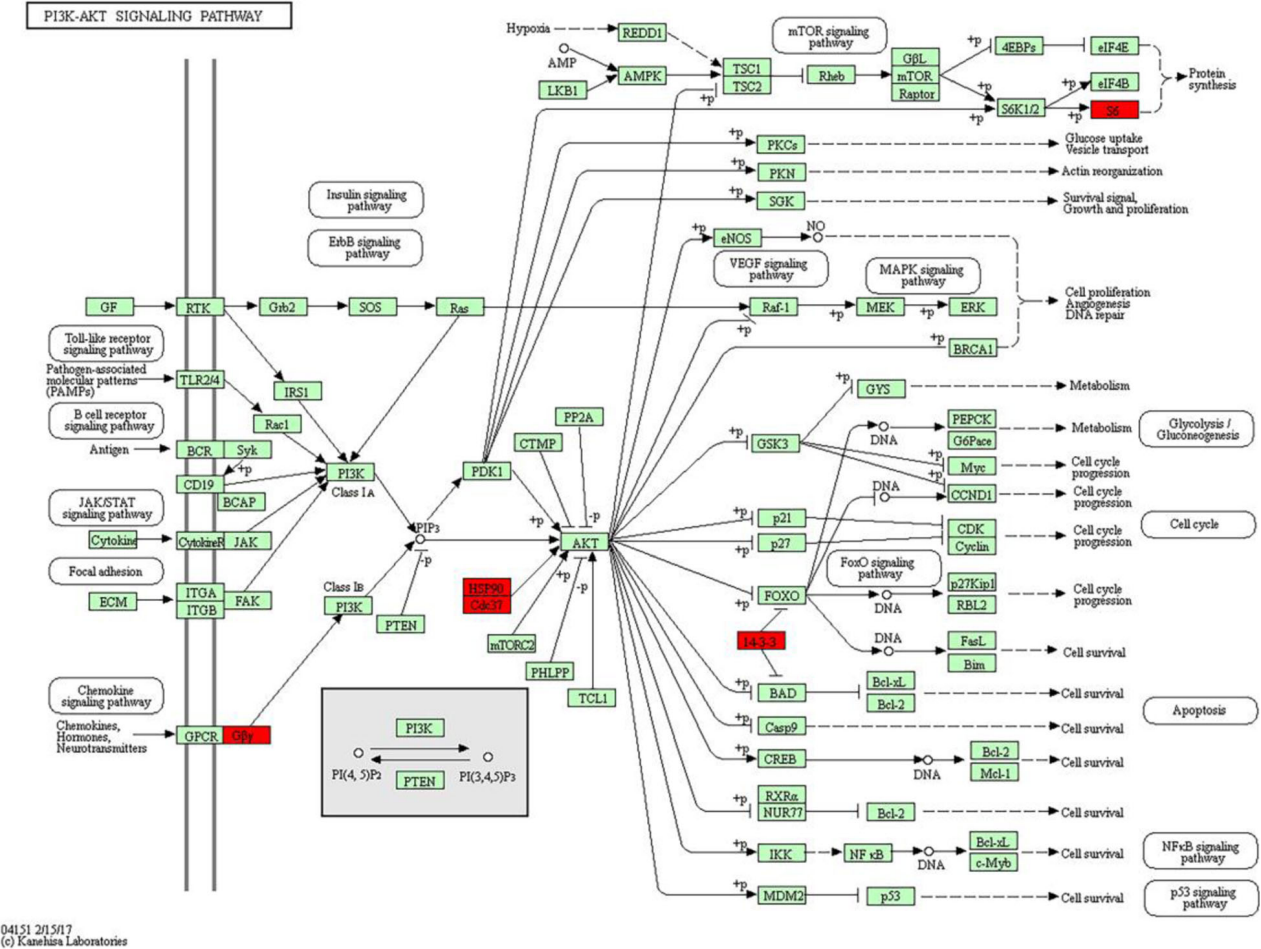
PI3K-Akt signaling pathway. Red represents differentially expressed proteins, 14–3-3 proteins family (YWHAG, YWHAH, YWHAB, YWHAQ), HSP90AB1, CDC37, GNG10 and RPS6 exist in PI3K-Akt signaling pathway

One of the main uses of the GO is to perform enrichment analysis on protein sets. Total 3409 proteins were identified from the 30,321 peptides sequences obtained from LC-MS/MS (FDR ≤0.01) (Additional file 1: Table S1). Out of 3409, 259 were differentially expressed including 26 upregulated proteins (fold change ≥2) and 233 downregulated proteins (fold change ≤0.5) in DF (Additional file 2: Table S2). The differentially expressed proteins were categorized under 3 major GO classifications: biological process, cellular component and molecular function (Additional file 3: Table S3). Among the biological processes, many biological processes were associated with follicular development such as cellular process (17.55%), metabolic processes (14.78%), regulation of biosynthetic process (9.31%) and cell death (1.55%) (Fig. 1). With respect to their molecular function, most of the proteins were involved in nucleic acid binding (20.04%) and protein binding (17.89%) (Fig. 2). Regarding cellular components, most of the proteins were assigned to the nucleus (11.46%) and cytoplasm (18.69%) (Fig. 3).

**Fig. 4:**
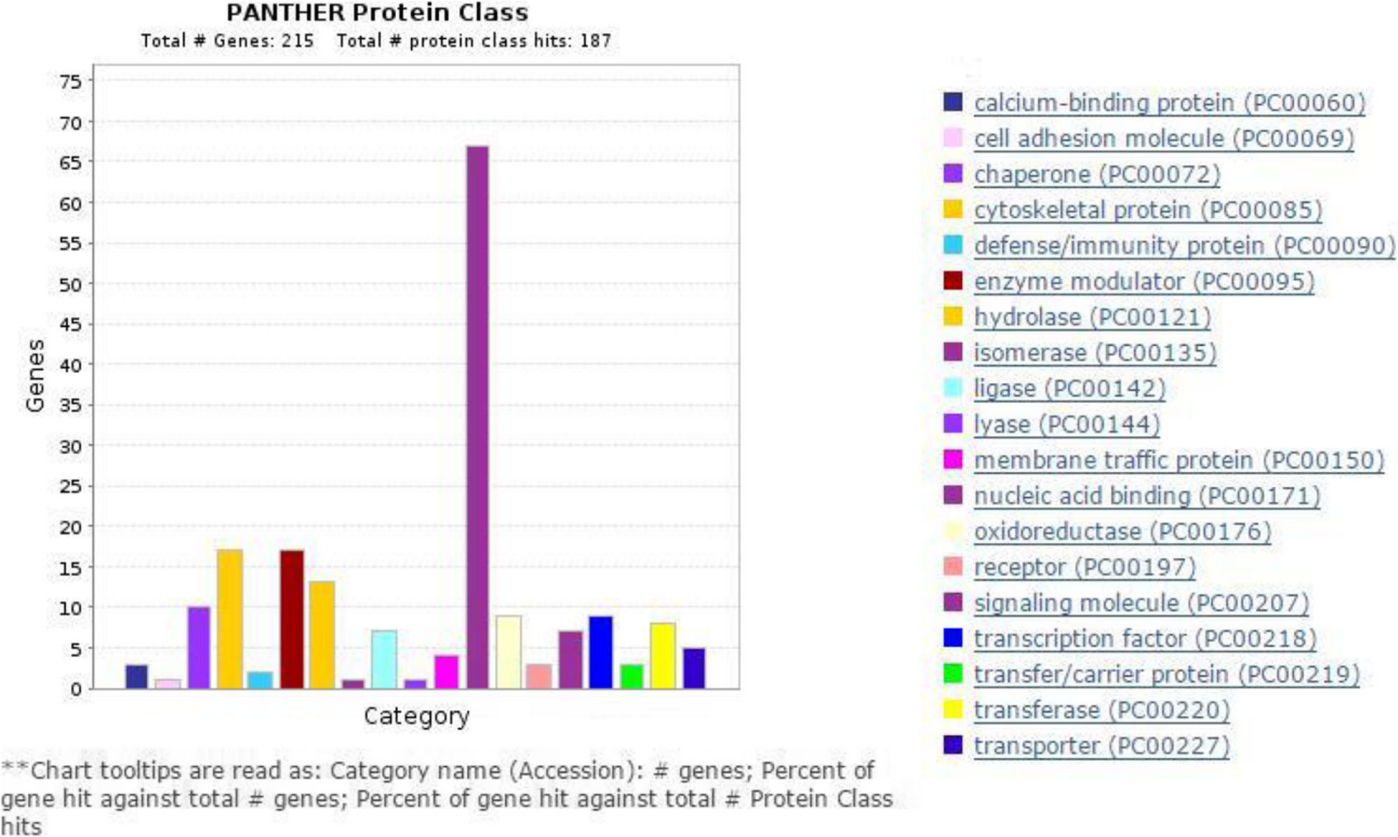
Differentially expressed proteins COG analysis. Different functions are displayed by different colors, x-coordinate represents category names, y-coordinate represents protein amount

### KEGG pathway analysis

KEGG pathway analysis results showed the most important biochemical metabolic pathways and signal transduction pathways were associated with follicular development (Additional file 4: Table S4). such as estrogen signaling pathway, oocyte meiosis, progesterone-mediated oocyte maturation, apoptosis signaling pathway, insulin signaling pathway and p53 signaling pathway, etc. Especially, PI3K-Akt signaling pathway is classical signaling pathway in follicular development regulation, including 8 differentially expressed proteins in PI3K-Akt signaling pathway (HSP90AB1, GNG10, YWHAG, YWHAH, YWHAB, YWHAQ, RPS6, CDC37) (Additional file 5: Figure S1). Also, HSP90AB1 is involved in estrogen signaling pathway and progesterone-mediated oocyte maturation, RPS6 involved in mTOR signaling pathway.

### COG analysis

PANTHER was used to categorize differentially expressed proteins as different functional contents, and annotated set of 215 bovine genes as a reference dataset. 187 differentially expressed proteins were categorized for 19 functional contents through COG (Fig. 4), which are significant functional contents in follicular development such as, membrane traffic protein, receptor, signaling molecule and transcription factor (Additional file 6: Table S5).

### Correlation analysis between proteome and transcriptome

**Fig. 5:**
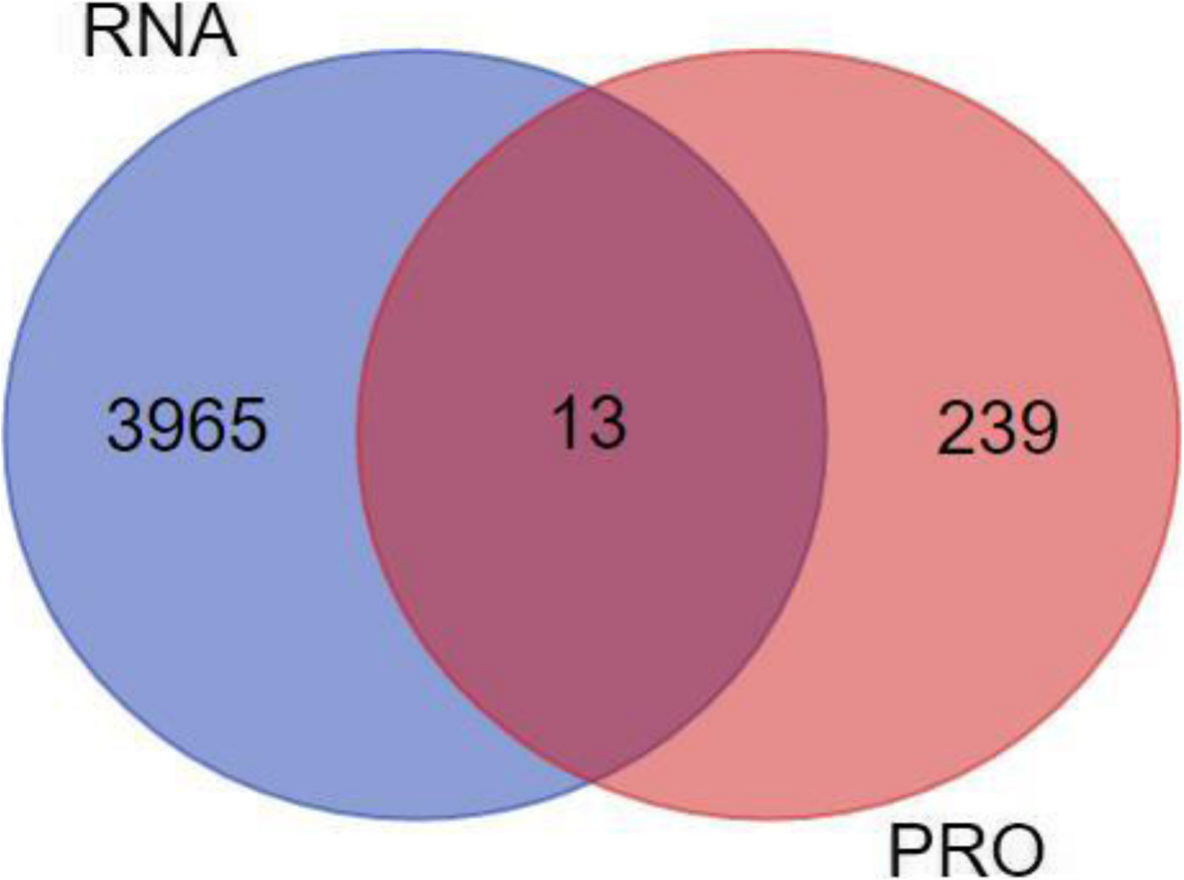
Venn diagram. Venn diagram of the overlapping significant upregulated or downregulated proteins shared among transcriptome and proteome

Correlation analysis was carried out by gene name, and proteins without gene name were not involved in the analysis. 15,520 genes (bovine RefSeq database containing 35,325 annotated transcripts) were obtained from the RNA-seq (RPKM cut off ≥0.5) (Additional file 7), of which 3978 differentially expressed genes were selected by GEO database (fold change ≥2), integrated into proteome data to further identify proteins of consistent expression profile by Venn diagram (Fig. 5). In total, 13 genes overlapped for their mRNAs and proteins expression status and other genes were expressed either in the form of transcript or protein only (Additional file 8).

### Changes of proteome profiles

**Fig. 6:**
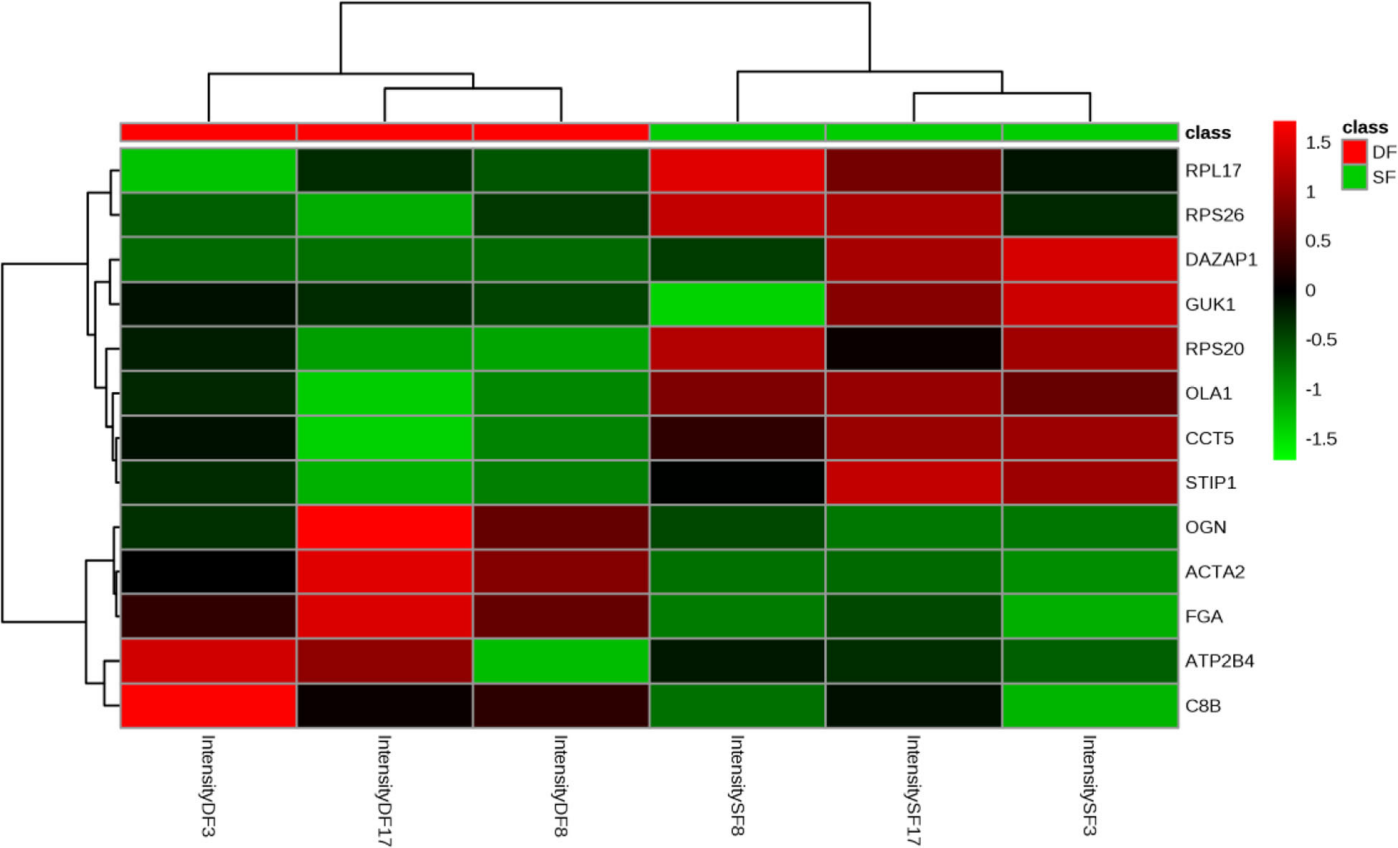
Heatmap. The heatmap presents relative abundance of the proteins with different colors, where deeper green represents lower intensity and deeper red represents higher intensity. Different Samples are displayed as columns and classified by proteomic subtypes as indicated by different colors. The gene names listed on the right of the heatmap

To compare the expression intensity of 13 differentially expressed proteins in DF and SF, a heatmap was applied (Fig. 6). The heatmap provides visual information of the data, where follicle samples were displayed as columns and classified as indicated by red color and green color, the abbreviations of 13 differentially expressed proteins listed on the right of the heatmap, sequential palettes fix the lowest and the highest value of proteins expression. These results elucidated 8 proteins (RPL17, RPS26, DAZAP1, GUK1, RPS20, OLA1, CCT5, STIP1) highly expression in SF, lowly expression in DF, while 5 proteins (OGN, ACTA2, FGA, ATP2B4, C8B) highly expression in DF, lowly expression in SF.

### Immunoblot validation

The expression tendency of three proteins were consistent with MS data.

## Discussion

This study represents the first investigation of multi-omics correlation analysis of bovine follicles and identified numerous new candidate proteins potentially interacting with mechanism of follicular development.

We found that specific highly differentially expressed proteins in DF, including fibrinogen (e.g.,FGA, FGB, and FGG), OGN and ACTA2, etc. FGA, OGN and ACTA2 are also 3 proteins of 13 differential associated proteins. Fibrinogen is an essential component of hemostatic system, normal pregnancy is associated with hypercoagulable state, fibrinogen increased progressively throughout pregnancy [24], it is suggested that fibrinogen might be a critical factor in follicle. OGN and ACTA2 are associated with tumors, and both inhibit cell proliferation. OGN expression in DF is highest compared with SF in 26 upregulated proteins, previous studies have shown loss of OGN expression in cancer cell lines and different tumors [25], suggesting its potential role as a tumor-suppressor gene. ACTA2 has positive effects on metastatic potential, it could be a promising therapeutic target for metastatic lung adenocarcinoma [26]. Cattle undergoes 2–3 follicular wave during one estrous cycle, only the DF of last follicle wave eventually ovulation. In this work, DF was selected from the first follicle wave, so DF also undergoes atresia. It is likely that OGN and ACTA2 are associated with anovulatory DF.

We also identified specific highly differentially expressed proteins in SF compared with DF, including 14–3-3 proteins (e.g., YWHAG, YWHAH, YWHAB, YWHAQ), ROR2, PA2G4, DAZAP1, OLA1, eukaryotic translation initiation factors (e.g.,EIF2S3, EIF4A1, EIF1, EIF3I, EIF5A, EIF2A, EIF4G2, EIF3J, EIF1B), DDX1 and CCT6A. 14–3-3 proteins, are considered as anti-apoptotic and critical regulatory proteins in cell division and apoptosis, might also play an important role in regulating cellular activities by associating with cytoskeletal proteins [27]. ROR2 expression is significantly increased in ovarian cancer [28]. PA2G4 is shown to regulate cell differentiation and growth [29]. DAZAP1, a ubiquitously expressed protein, is essential for the normal growth and development of mice [30]. OLA1 is a translational regulator of p21, which has an important impact on promoting cell proliferation [31]. Accumulatied evidence suggests that eukaryotic initiation involves a large number of eukaryotic initiation factors [32–34], 9 eukaryotic translation initiation factors and ATP-dependent RNA helicase DDX1 were found in proteome data**,** which are correlated with translation initiation [35] and cell proliferation [36]. It is suggested that14–3-3 protein family (YWHAG, YWHAH, YWHAB, YWHAQ), ROR2, PA2G4, DAZAP1, OLA1, eukaryotic translation initiation factors and DDX1 are likely to be important proteins in cell proliferation. CCT6A likely plays important role in follicle growth and sexual maturity in hens [37]. In GCs isolated from pre-ovulatory follicles, progesterone activated CCT6A, whereas follicle-stimulating hormone (FSH) inhibited the expression of CCT6A mRNA [38]. FSH targets GCs exclusively to induce their differentiation and maturation [39], the onset of each follicular wave is preceded by FSH transient increase, whereas DF selection occurs in the face of declining FSH concentrations [40], so CCT6A expression level is lower in DF. Unequivocally, these downregulated proteins were associated with cell growth. It’s well known that SF finally developed into atresia follicle, paradoxically, highly expression of these proteins could lead to GCs proliferation, which needs to be further research.

GO analysis of differentially expressed proteins showed most of the proteins distributed in cytoplasm of the cell, and involved in nucleic acid binding and protein binding, and associated with metabolic process, macromolecule metabolic process, biosynthetic process, regulation of biological process. Therefore, it appears that most of these proteins are involved in regulation of metabolism.

To clarify the metabolic pathways in which these proteins were involved, we performed KEGG pathway analysis, 46 signaling pathway are listed in Fig. 2, of these are associated with follicular development and possess differentially expressed proteins, such as PI3K-Akt signaling pathway, estrogen signaling pathway, progesterone-mediated oocyte maturation, mTOR signaling pathway, apoptosis, wnt signaling pathway, insulin signaling pathway, metabolic pathways. It is known that Wnt signaling is essential for embryonic development and cellular processes [41], insulin is crucial for GCs function, follicle growth and ovulation in cows [42], PI3K-Akt pathway plays an important role in follicular development, estradiol initiate estrous behavior, which is critical to sustain DF growth [43], etc. This also verify the accuracy of proteome results. Interestingly, 14–3-3 proteins (e.g., YWHAG, YWHAH, YWHAB, YWHAQ) were not only highly differentially expressed proteins in SF, but enriched in both PI3K-Akt signaling pathway and Hippo signaling pathway. Therefore, YWHAG, YWHAH, YWHAB, YWHAQ may be the primary proteins responsible for follicular development. Oxidative stress may be related to folliculogenesis and oogenesis [44] in the bovine species, SOD2 – mitochondrial can act directly on superoxide anion radicals, especially, estradiol inhibited SOD2 mRNA expression in rat luteal cells [45]. KEGG analysis showed that SOD2 participate in FoxO signaling pathway, and SOD2 was upregulated in DF, this may indicate that SOD2 may play an important role in DF.

In the current study, most genes that differentially expressed in proteome data, whereas not differentially expressed in transcriptome data, so only 13 differentially expressed proteins were identified through omics association analysis. Furthermore, regulation of mRNA turnover in the cell is essential for controlling the abundance of cellular transcripts, in turn, the levels of protein expression [46, 47]. mRNA half-life is linked to the function of the encoded protein, substantial stability of a mRNA means that it will be available for translation for a longer time, leading to high levels of protein gene products. mRNAs of many regulatory genes, which encode proteins that are required for only a short time—such as regulators of growth or differentiation, cell cycle regulators—often have short half-lives [48], so mRNAs of some proteins might not be detected because of mRNAs degradation, such as ELOC and SERPINA3 are differentially expressed proteins, which RNA were not found in transcriptome data. Recent studies revealed that ribosomal proteins (RPs) have been associated with cell proliferation, survival, apoptosis and other cellular processes, such as RPS20 was able to increase cell cycle arrest and cell death, and extend the half-life of p53 by more than 4-fold [49]. 31 RPs are all down-regulated in proteome data, and specifically, the expression of RPS26, RPL17, RPS20 decrease in both transcript and protein levels, It is suggested that RPS26, RPL17, RPS20 possess stable functions and may play an important role in the follicular development.

## Conclusions

The present study characterized the GCs proteome of bovine follicles at specific stages by a label-free strategy, a total of 259 proteins were identified as differentially expressed proteins (fold Change > 2, *P* < 0.05), 13 differentially expressed proteins were identified in DF versus SF by performing translatomics integrated with proteomics analysis, some proteins of 259 differentially expressed proteins may be associated with follicular development, including eukaryotic translation initiation factors (e.g.,EIF2S3, EIF4A1, EIF1, EIF3I, EIF5A, EIF2A, EIF4G2, EIF3J, EIF1B), 14–3-3 proteins (e.g., YWHAG, YWHAH, YWHAB, YWHAQ), fibrinogen (e.g., FGB and FGG), etc. Some proteins whose mRNA are absent because of short mRNA half-life. Bioinformatics analyses also provided metabolic pathway information about these proteins, however, much more research is needed to confirm that these proteins are biomarkers for follicular development.

## Supplementary information

Additional file 1: **Table S1.** List of proteins (3409) expressed in DF and SF from the 30,321 peptides (FDR ≤ 0.01).

Additional file 2: **Table S2.** List of genes (15,520) expressed in DF and SF follicles with a cut-off RPKM of 0.5. **Table S2.** List of 259 differentially expressed proteins in DF and SF (Fold Change > 2, *P* < 0.05).

Additional file 3: **Table S3.** List of Go analysis of 259 differentially expressed proteins.

Additional file 4: **Table S4.** List of KEGG pathway analysis of 259 differentially expressed proteins.

**Additional file 5.** Eight differentially expressed proteins in PI3K-Akt signaling pathway.

Additional file 6: **Table S5.** List of COG analysis of 259 differentially expressed proteins.

Additional file 7: **Table S6.** List of genes (15,520) expressed in DF and SF follicles with a cut-off RPKM of 0.5.

Additional file 8: **Table S7.** List of 13 differentially expressed genes in DF and SF.

## Data Availability

Availability of data and materials are included in the manuscript, figures and tables.

## Abbreviations

AGC: Automatic gain control
CID: Collision-induced dissociation
COG: Cluster of Orthologous Groups
DF: Dominant follicle
FDR: False discovery rate
FSH: Follicle-stimulating hormone
GCs: Granulosa cells
GO: Gene ontology
IGF-1: Insulin-like growth factors 1
LC-MS/MS: Label-free liquid chromatography-tandem mass spectrometry
PGF2α: Prostaglandin F2α
RPs: Ribosomal proteins
SF: Subordinate follicle
VEGF: Vascular endothelial growth factor
