# Supplementary figures and images for: Unraveling proteome changes and potential regulatory proteins of bovine follicular Granulosa cells by mass spectrometry and multi-omics analysis
Source: Proteome Sci. 2019 Oct 25;17:4. doi: 10.1186/s12953-019-0152-1 (PMC6815045; doi:10.1186/s12953-019-0152-1)

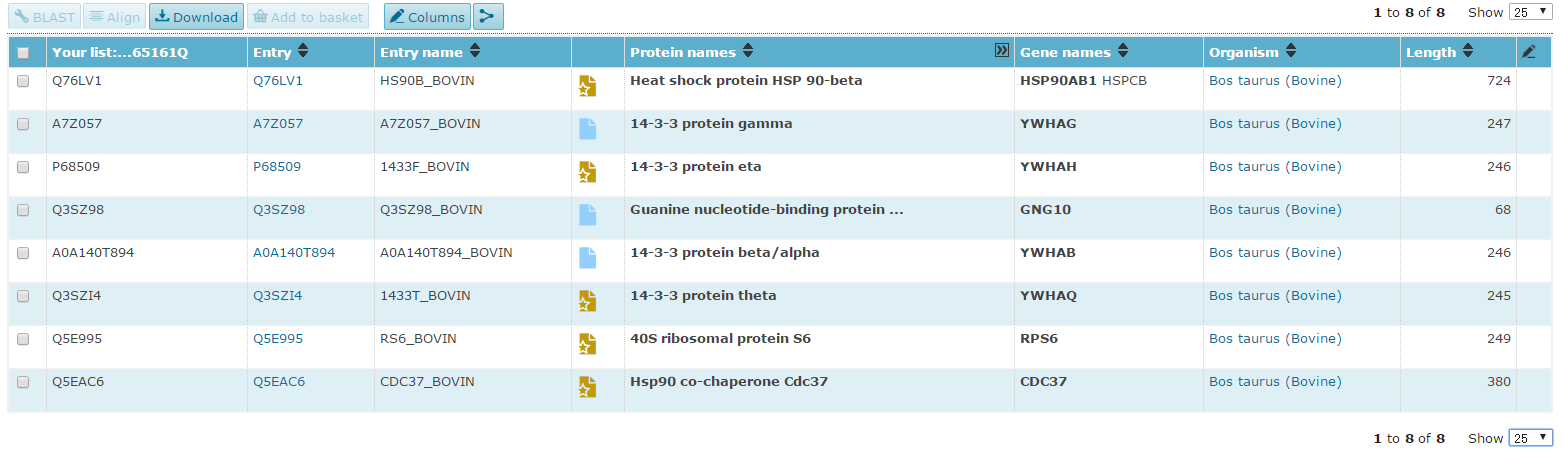

Supplement: Supplementary file 5 — Additional file 5. Eight differentially expressed proteins in PI3K-Akt signaling pathway. [file 12953_2019_152_MOESM5_ESM.png]
